# Impact of Agile Learning on Innovative Behavior: A Moderated Mediation Model of Employee Engagement and Perceived Organizational Support

**DOI:** 10.3389/fpsyg.2022.900830

**Published:** 2022-06-21

**Authors:** Yunseong Jo, Ah Jeong Hong

**Affiliations:** ^1^Social Science Korea Research Team, Chung-Ang University, Seoul, South Korea; ^2^Department of Education, Chung-Ang University, Seoul, South Korea

**Keywords:** learning agility, employee engagement, perceived organizational support (POS), innovative behavior, moderated mediation effect

## Abstract

This study analyzed learning agility, employee engagement, perceived organizational support (POS), and innovative behavior related to the development of innovative environment and the mental and psychological health of employees. A substantial body of research has examined the antecedents of innovative behavior of employees in their work environment, but our current understanding of how learning and motivational aspects of employees synthetically influence the innovative behavior remains incomplete. To address this gap, we developed and tested a moderated mediation model of the relationship between learning agility and employee engagement, POS, and innovative behavior. Following the job-demand resource model, componential theory, and social exchange theory, our postulated model predicted that the mediating effect of employee engagement on the relationship between learning agility and innovative behavior would be moderated by POS. The result of the analysis of the data on 331 corporate employees in South Korea supported this model. Specifically, learning agility was related to innovative behavior, while employee engagement mediated the relationship between learning agility and innovative behavior; POS strengthened the positive effect of learning agility on innovative behavior via employee engagement. We also discuss the implications of the results, future direction, and limitations of this study based on these findings.

## Introduction

In the era of a fast-growing and competitive knowledge-based economy, innovation is critical for an organization’s competitive advantage and sustainable performance (Chatzoglou and Chatzoudes, 2017). As the employees of the organization eventually perform the innovation, individual innovation behavior is a prerequisite for successful organizational innovation (Scott and Bruce, 1994). Various empirical studies have clearly identified the benefits of better employee organizational behaviors, which are considered an important source of an organization’s competitive advantage (Anderson et al., 2014; Harari et al., 2016). In contrast, failures in innovative behavior can lead to losses for both the organization and its employees (Tian et al., 2020). Therefore, it is important to study the antecedents and mechanism of the factors that facilitate innovative behavior and understand how these are generated.

Innovative behavior is influenced by individual learning characteristics, such as learning goal orientation (Montani et al., 2014) and motivation to learn (Yu et al., 2018), as well as by organizational factors, including learning organization (Park et al., 2014) and learning climate (Cangialosi et al., 2020). Thus, learning that promotes innovative behavior is an important factor. According to the componential framework of creativity, domain-relevant skills include knowledge, technical skills, expertise, and special talents that provide essential background knowledge and a basis for innovation (Amabile, 1983). Amabile (1983) contends that the larger the domain-relevant skillset, the more the alternatives for innovation. While innovative behavior is an individual-level behavioral aspect, learning agility, which is also a characteristic of individual learning, predicts innovative behavior.

Due to the complexity and unpredictability of the current business environment, employees need to have learning agility to constantly acquire new skills and learn new ways of performing their job (Milani et al., 2021). However, despite their importance, the consequences of learning agility in organizations have been rarely explored. Employees with high learning agility are characterized as risk-taking, open-minded, and tolerant, and accepting of challenges and innovation (Eichinger et al., 2010). Learning agility is closely related to perseverance in the face of ambiguity, risk preference, and flexible thinking; it induces innovative behavior (Farr and Ford, 1990). Accordingly, we hypothesize that the learning agility of employees affects their innovative behavior. From the componential theory perspective, innovative behavior should concurrently consider learning aspects such as learning agility and motivation for performing the task. Nevertheless, previous studies did not comprehensively examine these factors, but indicate only a simple relationship between learning agility and innovative behavior (Han, 2018; Kwon and Lee, 2020; Chu and Kim, 2021; Putri and Suharti, 2021). Thus, there is a need to explore the mechanism between learning agility and innovative behavior.

This study attempts to explore the process, the journey from learning to employee innovation, in a business context. First, we propose that employee engagement plays a mediating role in the relationship between learning agility and innovative behavior. In componential theory, organizational members’ creativity requires both domain-relevant skills and task motivation. In other words, to properly generate employees’ innovative behavior, a high level of motivation related to their task must be considered in conjunction with learning agility. From this perspective, employee engagement, the optimal work-related motivational state, is considered an important resource predicting innovative behavior. When employees are engaged in their work, they feel positive and are able to broaden their cognitive and behavioral repertoire, leading to creative and innovative behavior (Fredrickson, 2001). Previous studies have shown that employee engagement significantly affects their innovative behavior (Kwon and Kim, 2020).

Next, in terms of the relationship between learning agility and employee engagement, learning agility may predict employee engagement. Learning-oriented employees are more engaged in their jobs; they find challenging activities that arouse interest and curiosity, reflect on themselves, and engage in exploratory learning (Dweck, 1986; Dweck and Leggett, 1988; Matsuo, 2019). The agility aspect included in learning agility is also associated with employee engagement. A survey of 22 organizations in six sectors showed that organizations with high agility had 20–30% higher employee engagement than those that did not. Employees with high agility are likely to develop a strong sense of autonomy, mastery, and purpose, which are associated with employee engagement (Mckinsey, 2021).

Along with exploration of knowledge and information through learning agility and task motivation through employee engagement, the work environment is a key element for building creativity and innovative behavior (Amabile, 1983). Perceived organizational support (POS) can be considered an environmental factor; it refers to employee perception of how much the organization values their contributions and cares about their well-being (Eisenberger et al., 2001). Prior studies have revealed that POS is one of the most strongly predictable innovative behavior of an employee (Yildiz et al., 2017; Qi et al., 2019; Fan et al., 2022). According to social exchange theory (SET), when employees believe that they are being encouraged, they tend to yield returns for their organization by putting in extra effort, such as by demonstrating innovative behavior (Eisenberger et al., 1990; Nazir et al., 2018). As motivation is a condition that determines the direction and intensity of a particular behavior, behavior can be strengthened by organizational support. Therefore, this study assumes that the relationship between employee engagement and innovative behavior is moderated by POS. As employee engagement depends on learning agility, this study identifies the moderated mediation effect whereby employee engagement influenced by learning agility regulates the mediation effect on the innovative behavior.

Few studies have examined the logical connection between learning agility, employee engagement, POS, and innovative behavior. If employees’ innovative behaviors are to be systematically enhanced and developed within an organization, a framework that aligns with the learning, engagement, and organizational support system is necessary. Therefore, the purpose of this study is to systematically investigate the relationship between learning agility, employee engagement, POS, and innovative behavior. In view of an increasing need for innovation, the results of this study could facilitate a better understanding of how employees’ innovative behavior can be enhanced.

## Learning Agility and Innovative Behavior

Organizations recognize the significance of employees’ innovative behavior as an intangible asset that produces the best ideas to stay competitive, regardless of task categories or the organization’s hierarchical standard. Innovative behavior and the process that motivates such behavior is an area of critical importance in business (Yuan and Woodman, 2010; Riaz et al., 2018).

Innovative behavior refers to an employee’s intentional introduction and application in a role, group or organization of ideas, processes, products, and procedures (West and Farr, 1989). Janssen (2000) defined innovative behavior as the intentional creation, introduction, and application of new ideas within a work role, group, or organization to benefit role performance, the group, or the organization. Examples of such behavior include looking for new technologies, suggesting new ways to achieve goals, applying new work processes, and investigating and securing resources to implement new ideas (Yuan and Woodman, 2010). Innovative behavior leads to various positive outcomes at the organizational and individual levels, including job productivity (Chang and Liu, 2008), task performance (Aryee et al., 2012), service innovation performance (Li et al., 2019), and firm growth (Stenholm, 2011). Innovative behavior has attracted substantial attention in the practical and academic fields.

As learning through experience is regarded as a way of improving productivity in uncertain market environments, learning agility can be one of the most important competencies. According to Lombardo and Eichinger (2000), learning agility is “the willingness and ability to learn new competencies in order to perform under first-time, tough, or different conditions” (p. 323). Researchers have discussed learning agility and learning orientation as different concepts. Learning orientation reflects a dispositional trait to expand the current knowledge set continuously (Dweck, 1986; De Rue and Wellman, 2009), while learning agility is a comprehensive concept and includes learning orientation and ability (Mettl, 2022). Previous empirical studies found that learning orientation could be an antecedent to learning agility (Drinka, 2018; Putri and Suharti, 2021).

Experiential learning theory, which supports learning agility, explains that the experiences of the members of an organization can be used as resources for new learning. In other words, organizational members can reconfigure experience to meet the goal and vision of the organization, or convert it into the skills and knowledge required for the job. Individuals with high levels of learning agility tend to seek new opportunities constantly, actively seeking feedback from others to grow and develop, and are likely to self-reflect, evaluate, and draw practical conclusions from their experiences (De Meuse et al., 2010). Companies with a high level of learning agility in their workforce consistently outperform competitors in terms of profitability, market share, sales growth, and customer satisfaction (Gravett and Caldwell, 2016). However, the procedural mechanism whereby the psychological characteristics and behaviors that form part of the learning agility of organizational members generate performance remains a mystery.

People with high learning agility pursue innovation without fear of new challenges, have high experimental tendencies, and produce results through communication with others (Eichinger et al., 2010). These characteristics of learning agility are directly related to the innovative behaviors that explore different ideas, find new methods, communicate with others to apply them to organizations, and produce results through execution (Janssen, 2000). Staw (1990) proposed problem identification and resolution, creativity, and communication skills as the basis for innovative behavior. These factors refer to the individual’s inclination toward innovative behavior, acceptance of challenges and newness, communication with others, flexible thinking, and the will to create results, and are closely related to the components of learning agility suggested by Eichinger et al. (2010). Specifically, mental agility, which is characterized by curiosity and comfort with ambiguity and complexity; people agility, which is related to open-mindedness, flexible attitudes, and communication skills; change agility, which includes experimentation, trying new things, and easily accepting challenges; and result agility, which relates to creating results, can serve as major resources for innovative behaviors. According to the individual adaptability theory, which describes the learning agility of organizational members, innovative behavior is a result of the learning agility of members within the organization. Recent empirical studies also suggest that learning agility of organizational members is related to innovative behavior (Han, 2018; Kwon and Lee, 2020). Based on this discussion, we formulate the following hypothesis.

H1: Learning agility is positively related to innovative behavior.

## The Mediating Role of Employee Engagement

Positive psychology, which emerged in the 2000s, is acclaimed as an alternative approach centered on human strengths and optimal functioning, an outgrowth of the negative psychology approaches studying the traditional four Ds: disease, damage, disorder, and disability (Diener et al., 1999; Luthans and Avolio, 2009). Accordingly, engagement came to be understood as the converse of burnout, a state of negativity in relation to work. Employee engagement refers to “a positive, fulfilling, work-related state of mind that is characterized by vigor, dedication, and absorption” (Schaufeli et al., 2002, p. 74). Shuck et al. (2017) defined employee engagement as “an active, work-related positive psychological state operationalized by the intensity and direction of cognitive, emotional, and behavioral energy” (p. 959). Concerning the terms, although employee engagement, work engagement, and job engagement are often used interchangeably by scholars and practitioners (Shuck and Wollard, 2010), the term “employee engagement” has recently gained preference (Rothwell, 2014; Shuck et al., 2017). Accordingly, employee engagement is considered a positive psychological state with cognitive, emotional, and behavioral components associated with job and organization. Each component of employee engagement is described as follows (Macey and Schneider, 2008; Shuck et al., 2017): Cognitive engagement is the intensity of mental energy expressed toward positive job and organizational outcomes. Emotional engagement is the intensity and willingness to invest emotionally in positive job and organizational outcomes. Behavioral (physical) engagement is the psychological state of intention to behave in a manner that positively affects both in-role and extra-role performance.

Empirical evidence reveals that employee engagement is negatively related to psychosomatic health complaints (Schaufeli and Bakker, 2004) and positively to psychological/mental health (Hakanen and Schaufeli, 2012; Torp et al., 2013), physical health (Seppälä et al., 2012; Rongen et al., 2014), and happiness in the workplace (Bakker and Oerlemans, 2016). Furthermore, employee engagement facilitates the use of cooperative interpersonal tactics, reduces workplace conflict, and creates a positive work environment and climate (Demerouti and Cropanzano, 2010). In sum, employee engagement is considered a key factor in building positive and innovative workplaces as well as enhancing employees’ physical and psychological health.

According to Kwon and Kim’s (2020) integrative literature review, employee engagement is an important positive determinant of innovative behavior. Employee engagement is expected to drive innovative behavior developed from the synergy of cognitive, emotional, and physical energies (Hakanen et al., 2008). Specifically, cognitive engagement stimulates innovative behavior by allowing employees to revisit their experience and knowledge structure, broaden the scope of cognition and perception, try various suggestions, and generate new ideas (Fredrickson, 2001). Emotional engagement allows employees to feel confident in the purpose and meaning of innovative efforts, to be optimistic about innovation, and to help fuel proactive behavior across the organization (Demerouti and Cropanzano, 2010; Shuck et al., 2017). Physical engagement is a determinant in overcoming stress and fatigue in the process of innovation, realizing ideas, and maintaining innovative motivation and behavior (Kwon and Kim, 2020). When employees are engaged, they become proactive, show initiative, persist in the face of difficulties, effectively collaborate with others, and invest energy in their work (Leiter and Bakker, 2010). These behaviors are particularly relevant to innovative activities (Amabile, 1988; Zhang and Bartol, 2010; Chang et al., 2013). Numerous studies have demonstrated that employee engagement is related to innovative behavior (Agarwal, 2014; Gorgievski et al., 2014; Chen and Huang, 2016; Kim and Park, 2017).

Meanwhile, employee engagement depends on employees’ learning agility. Employees’ activities related to learning (e.g., opportunities for continuous learning, inquiry, and dialogue) play an important role in facilitating engagement through extrinsic as well as intrinsic motivational potential by assisting employees in achieving goals and facilitating growth (Eldor and Harpaz, 2016). In addition, learning experiences shape stronger positive self-evaluations and efficacy (Kohn and Schooler, 1982; Salanova et al., 2010). Efficacy is related to being fully absorbed in the task as well as to expending higher levels of energy and effort to complete a task, which results in employee engagement (Sweetman and Luthans, 2010). Based on these discussions, we infer that learning agility, the tendency to pursue continuous learning in the workplace, will affect employee engagement. Recent empirical research has shown that learning agility is related to employee engagement. Saputra et al. (2018) confirmed that the learning agility of employees in various industries, such as ICT, manufacturing, and service, has a direct effect on employee engagement. A study of outstanding nurse managers found that change agility is related to employee engagement (Mackoff and Triolo, 2008). Further, the results of various empirical studies have proven that learning agility has a significant and positive effect on employee engagement (Jeong and Sung, 2018; Saputra, 2018; Kwon and Lee, 2020).

Thus, employee engagement is both a result of learning agility and an antecedent to innovative behavior. As a mediator between learning agility and innovative behavior, employee engagement integrates learning and innovation into the psychological process, including cognition, affection, and behavior in the workplace. This mediating role of employee engagement was set, based on the Hobfoll’s (1989) conservation of resource theory, which is rooted in a resource maximization model in which an increase in resources leads to additional resource accumulation as a result of the so-called “gain spirals.” Resource surplus with employees keeps them engaged in their jobs to conserve the resources needed to achieve goals (Hobfoll et al., 2018). Based on conservation of resource theory, it can be argued that resources such as positive self-evaluation, efficacy, and esteem can be acquired through learning agility, which in turn increases employee engagement to acquire those resources and makes them perform innovative behaviors (Islam and Tariq, 2018). Based on the theoretical outline and empirical results mentioned earlier, we formulate the following hypothesis:

H2: Employee engagement mediates the relationship between learning agility and innovative behavior.

## The Moderating Role of Perceived Organizational Support

Based on the analysis described earlier, we proposed that learning agility predicts innovative behavior among employees through engagement. However, this mechanism may vary with organizational support. The theory of innovation allows us to postulate that an individual’s innovative behavior is a function of a continuous process of interaction between the individual and contextual influences (Woodman et al., 1993). Contextual influences are characterized by organizational support such as reward, caring, and recognition (Baran et al., 2012). When organizational support is sufficiently perceived, it can foster innovative behavior (Eisenberger et al., 1986).

Perceived organizational support refers to the degree to which employees believe their organization values their contributions and cares about their well-being (Eisenberger et al., 1986). According to SET, employees’ POS is formed in response to their socio-emotional needs, and the organization’s readiness to reward increases work efforts (Rhoades and Eisenberger, 2002). SET maintains that, based on the norm of reciprocity, employees trade effort and dedication with their organization for tangible incentives such as rewards and fringe benefits, and socio-emotional benefits such as esteem, approval, and caring (Baran et al., 2012). Numerous studies have found that employees with higher levels of POS feel more obligated and perform innovative behaviors that benefit the organization (Afsar and Badir, 2017; Nazir et al., 2018).

Perceived organizational support is also valued as an assurance that aid will be available from the organization when it is needed to deal with stressful situations [cf. George et al. (1993)]. The process of innovation is complex and strenuous, requiring considerable time and effort to see results (Anderson et al., 2014). According to Karasek’s (1979) demand-control model, innovation tends to lead to stress by increasing the mental demands employees face. Specifically, innovations always imply a degree of change and often uncertainty, which is usually experienced as a form of stress, triggering the individual to be alert and adjust to the changing circumstances (Cowan et al., 2011). In addition, there is abundant empirical evidence showing that innovation induces stress, fatigue, and burnout (Rafferty and Griffin, 2006; Chung et al., 2017; Hammond et al., 2019). POS, in contrast, prevents the negative consequences of innovation and promotes continuous innovative behavior.

An organization attends to its employees’ well-being, concedes contributions, and is susceptible to their needs because those interventions quickly lead to innovative behavior from the employee. Engaged employees can transform their own positive and proactive energy into innovative behaviors by recognizing that the organization they belong to cares for and supports them appropriately. POS can be considered a representative organizational resource; as an individual supplemental resource, POS can generate a range of positive emotional perceptions and experiences in the workplace (Rhoades and Eisenberger, 2002; Arnold and Dupré, 2012). According to broaden-and-build theory (Fredrickson, 1998), positive emotions serve to broaden individual thought–action repertoire, which in turn has the effect of building individuals’ physical, intellectual, and social resources to bring them indirect and long-term adaptive benefits. Compared with people who have positive emotion and enough resources, people who have negative emotion and experience a lack of resources are more sensitive to opportunity cost and perceive a higher level of psychological threats, which leads to conservative decision making (Zhang et al., 2017; Cho and Song, 2021). Thus, when engaged employees recognize appropriate organizational support, they display a higher level of innovative behavior.

Indeed, the literature shows that POS has shown a significant moderating effect on the relationship between engagement and innovative behavior. A study conducted on 220 employees in four innovative industries found that POS had a significant moderating effect on the relationship between cognitive engagement and innovative performance (Fachrunnisa et al., 2020). Yet another study conducted with 1,049 customer service employees showed that the interaction term between engagement and POS had a significant effect on task performance. In other words, POS has a decisive moderation effect on the relationship between engagement and task performance (Yongxing et al., 2017). Furthermore, POS has been confirmed to be an influential moderating variable that regulates employees’ innovative behavior (Yildiz et al., 2017; Yoon et al., 2019). The present study examined the moderating effect of organizational support on the relationship between employee engagement as individual-level variables and innovative behaviors. The main reason to choose this factor was to explore how organizational support effectively enhances innovative behaviors. Based on the theoretical background and empirical results, we formulate the following hypothesis.

H3: The positive relationship between employee engagement and innovative behavior is moderated by perceived organizational support.

**FIGURE 1:**
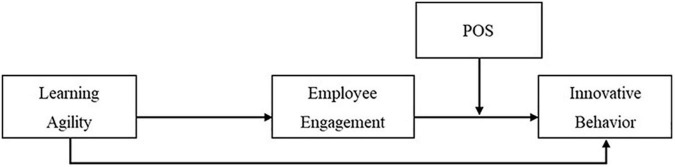
Theoretical moderated mediation model.

According to the job demand-resource (JD-R) model, a well-known theoretical framework for presenting the mechanism of employee engagement, employee engagement is facilitated by job resources and personal resources (Schaufeli and Bakker, 2004). Job resources are defined as aspects of the job that are functional in achieving work goals, reducing job demand, or stimulating personal growth and learning, such as learning opportunities (Halbesleben, 2010) and learning organization (Park et al., 2014). Personal resources are defined as aspects of the self that relate to the ability to control and affect one’s environment successfully, such as self-efficacy, optimism, and resilience. Learning agility is believed to serve as a representative personal resource for promoting employee engagement. The JD-R model assumes that, in its turn, engagement produces positive outcomes such as innovative behavior (Kwon and Kim, 2020). In other words, learning agility improves employee engagement and eventually raises the level of innovative behavior. Meanwhile, this model describes how the job demand factor moderates the mediation process (resource–engagement–outcome). Job demand is defined as aspects of the job that require sustained physical or mental effort, such as emotional demands and unfavorable work conditions (Schaufeli and Taris, 2014). POS lowers job demand by playing a role in alleviating the stress and fatigue arising from the job condition and increasing the level of innovative behavior among organizational members. In fact, in various studies, POS has been shown to regulate the negative impact of job demand, resulting in positive outcomes (Zacher and Winter, 2011; Jain et al., 2013; Du et al., 2018). Based on the theoretical outline and empirical results above, we formulate the following hypothesis(see Figure 1).

H4: Perceived organizational support moderates the relationship between learning agility and innovative behavior via employee engagement.

## Materials and Methods

### Participants

To achieve the research goal, online surveys were conducted among 350 employees of eight companies in South Korea, using a convenience sampling method. All participants were informed about the purpose of the survey, assured of the confidentiality of their responses, and made aware of their right to withdraw their consent to participate at any time. The study was conducted under the principles of the American Psychological Association on research ethics. To ensure survey data quality, 19 careless or incomplete responses were excluded. A final pool of 331 valid responses was analyzed.

Among the participants, 43.2% were male and 56.8%, female. In terms of age, 24.5% were under 29 years old; 50.5% were between the ages of 30 and 34, 13.0% were between 35 and 39 years, 6.9% were between 40 and 50 years, and 5.1% were over the age of 50. In terms of academic qualifications, 2.7% had a high school diploma, 10.3% had an associate degree, 74.0% had a bachelor’s degree, 1.2% had a master’s degree, 0.3% had a doctorate, and 0.3% were classified as others. In terms of industry type, 25.1% were in service firms, 21.5% were in manufacturing, 12.4% were in social overhead capital firms, 8.5% were in public institutions, 6.3% in distribution, and 26.3% were classified as others. In terms of job category, 23.6% were in management planning, 16.8% were in accounting, 14.4% were in production/service, 12.7% were in research and development, 6.8% were in marketing, 3.8% were in human resource management and development, and 21.9% were classified as others. In terms of position, 77.3% were at the associate level, 16.9% at the manager level, and 5.7% were at the director level. In terms of work experience, 45.0% of the participants had worked for less than 5 years; 37.2% had worked for 5–9 years; 9.7% had worked for 10–14 years; 3.6% had worked for 15–19 years; and 4.5% had worked for more than 20 years. In terms of organizational size, 56.8% were working at companies with fewer than 300 employees, 20.8% were working for companies with 300–999 employees, and 22.4% were working for companies with 1,000 employees.

### Measures

#### Learning Agility

Learning agility was the independent variable for this study. Developed by Bedford (2011), the learning agility scale consists of six items, including “I reflect on and learn from mistakes.” Responses were measured by a five-point Likert scale ranging from 1 (strongly disagree) to 5 (strongly agree). The Cronbach’s alpha coefficient was 0.67.

#### Employee Engagement

The employee engagement scale for measuring employee engagement was developed by Shuck et al. (2017). It consists of 12 items and three dimensions (i.e., “emotional engagement,” “behavioral engagement,” and “cognitive engagement”). Emotional engagement includes four items such as “I feel a strong sense of belonging to my job.” Behavioral engagement includes four items such as “I really push myself to work beyond what is expected of me.” Cognitive engagement includes four items such as “I concentrate on my job when I am at work.” A five-point Likert-type scale was used. The Cronbach’s alpha coefficient was 0.90.

#### Perceived Organizational Support

The POS scale was derived by Eisenberger et al. (1986) and revised by McMillin (1997). The scale contains 10 items across two dimensions: emotional support (five items such as “My organization really cares about my well-being”) and instrumental support (five items such as “If the organization could hire someone to replace me at a lower salary it would do so [Reverse item]”), measured using a five-point Likert scale. The Cronbach’s alpha coefficient was 0.85.

#### Innovative Behavior

We assessed innovative behavior using six items developed by Scott and Bruce (1994), for example, “I search out new technologies, processes, techniques, and/or product ideas.” A five-point Likert scale was used. The Cronbach’s alpha coefficient was 0.87.

#### Control Variables

Respondent demographics, gender (Mikhailova and Kaminskaya, 2016), working years (Leong and Rasli, 2014), position (Liu et al., 2016), and organization size (Link and Bozeman, 1991) were used as control variables in this research, as these play an important role in increasing innovative behavior.

### Data Analyses

Data analyses were conducted using SPSS Statistics v.25, SPSS AMOS v.25, the SPSS PROCESS macro by Hayes (2015). SPSS Statistics was employed to calculate descriptive statistics and Pearson’s correlation matrix of all study variables. Confirmatory factor analysis (*CFA*) was conducted with IBM SPSS AMOS, and Hu and Bentler’s (1999) indices guideline were used to evaluate the goodness-of-fit indices. As a rule of thumb, *TLI* and *CFI* ≥ 0.90, and *RMSEA* and *SRMR* ≤ 0.08 were considered indicative of good model fit (Byrne, 2013). The moderated mediation model was tested using Model 14 in the SPSS PROCESS macro. Regression coefficient and bias-corrected 95% confidence intervals (CIs) were calculated using a bootstrapping procedure (5,000 re-sampling). The moderation variable was mean-centered. All statistical significance was determined at *p* < 0.05.

In this study, item parceling was used to aggregate individual items into sub-dimension for employee engagement and POS, which reflect multi-dimension, before conducting *CFA*. Item parceling is a technique used to create an aggregate-level variable comprising average of the individual items (Little et al., 2002). Using item parceling in CFA has been recommended because of advantages such as greater reliability (Kishton and Widaman, 1994), higher communality (Little et al., 2002), and less item-idiosyncratic influence (Chapman and Tunmer, 1995). Finally, “because aggregating items into parcels reduces the number of indicators involved in modeling, researchers are able to use more realistic models that better capture and more easily interpret increasingly complex theories of human behavior” (Nasser-Abu Alhija and Wisenbaker, 2006, p. 205). The overall fit of employee engagement (*x*^2^ = 151.241, *df* = 51, *TLI* = 0.94, *CFI* = 0.96, *RMSEA* = 0.08, *SRMR* = 0.50) and POS (*x*^2^ = 117.232, *df* = 34, *TLI* = 0.91, *CFI* = 0.93, *RMSEA* = 0.09, *SRMR* = 0.52) met the cut-off criteria. Therefore, the measurement model of these constructs was found to be statistically acceptable and all the individual items measuring these latent variables were formed into a parcel for each.

Furthermore, a *CFA* for the single common factor model was used to assess the common method bias (Podsakoff et al., 2003). The result of the *CFA* indicated that it fit poorly with the collected data (*x*^2^ = 599.872, *df* = 87, *TLI* = 0.69, *CFI* = 0.74, *RMSEA* = 0.13, *SRMR* = 0.09). As there is no single common factor explaining the major variance, common method bias is not considered a major problem in this study.

A power test was conducted using SPSS Sample Power v.3 to estimate the proper number of participants. To achieve a power of 0.80, with an estimate of a small effect size [*f*^2^ = 0.02; Cohen (1988)] for the covariates, main effects, interaction effects, and an alpha level of 0.05 (two-tailed), a sample of 378 was required. To achieve a power of 0.80, with an estimate of a medium effect size [*f*^2^ = 0.15; Cohen (1988)] for the covariates, main effects, interaction effects, and an alpha level of 0.05 (two-tailed), a sample of 38 was required. Although we did not have sufficient power to detect a small effect size, we did have sufficient power to detect a medium or large effect size.

## Results

### Validity and Reliability

A *CFA* was carried out for testing construct validity and reliability. To test for construct validity, we assessed for convergent validity and discriminant validity. The convergent validity was evaluated the magnitude and significance of the standardized factor loadings (*SFL*) and composite reliability (*CR*). The *SFL* values of the measurement model which were all above the threshold of 0.5 (Hair et al., 2018), *SFL* coefficients were between 0.51 and 0.92 after deleting low *SFL* value items. Furthermore, the t-values of SFL were significant. The *CR* values for all constructs ranged from 0.61 to 0.87, which exceeds the convergent validity threshold of 0.6 (Hair et al., 2018), and therefore, the convergent validity of the measure was appropriate.

The discriminant validity was measured by comparing the goodness-of-fit between different factor models (Rios and Wells, 2014). We conducted a series of *CFA*s to investigate whether all the variables examined in this study were distinct. When compared with other models, the proposed four-factor model structure (i.e., learning agility, employee engagement, POS and innovative behavior) was found to be a significantly better fit for the data (*x*^2^ = 191.590, *df* = 79, *TLI* = 0.93, *CFI* = 0.94, *RMSEA* = 0.07, *SRMR* = 0.06). This finding suggested that all the variables were distinct from one another. As an additional assessment of discriminant validity, we calculated several heterotrait-monotrait (*HTMT*) ratios of the correlations (Henseler et al., 2015), which is an alternative approach to the Fornell-Larcker criterion and the examination of cross-loadings and is based on the multitrait-multimethod matrix. Typically, when *HTMT* is over 0.85, there is a problem with discriminant validity (Henseler et al., 2015). As shown in Table 1, *HTMT* was calculated at 0.29–0.79, which shows that the constructs had adequate discriminant validity. In conclusion, the discriminant validity of the measure was at an appropriate level.

**TABLE 1:** Descriptive statistics, correlation, and heterotrait-monotrait (HTMT) matrix among study variables.

Variable	*M*	SD	1	2	3	4	5	6	7	8
1. Gender	0.57	0.50	−	−	−	−	−	−	−	−
2. Working years	0.85	1.04	–0.10	−	−	−	−	−	−	−
3. Position	0.28	0.56	–0.08	0.61**	−	−	−	−	−	−
4. Organizational size	0.66	0.82	−0.20**	0.01	–0.08	−	−	−	−	−
5. LA	3.89	0.58	−0.11*	–0.03	0.07	0.12*	−	*0.68*	*0.35*	*0.79*
6. EE	3.73	0.64	–0.09	0.08	0.15**	0.07	0.47**	−	*0.69*	*0.61*
7. POS	3.26	0.66	–0.08	0.15**	0.28**	0.01	0.22**	0.51**	−	*0.29*
8. IB	3.51	0.71	−0.17**	0.09	0.16**	0.02	0.59*	0.48**	0.22**	−

N = 331. Gender (0 = male, 1 = female); Working years (0 = under 5 years, 1 = 5–9 years, 2 = 10–14 years, 3 = 15–19 years, 4 = over 20 years); Position (0 = associate, 1 = manager, 2 = director); Organizational size (0 = under 300, 1 = 300–999, 2 = over 1,000). LA, learning agility; EE, employee engagement; POS, perceived organizational support; IB, innovative behavior; Values in italics denote a HTMT ratio. *p < 0.05, **p < 0.01.

For reliability, we measured Cronbach’s alpha (α) and *CR*. The α values for all constructs ranged from 0.67 to 0.90, which agreed with Nunnally’s criteria of 0.6 or above (Nunnally and Bernstein, 1994). *CR* values ranged from 0.61 to 0.87, which agreed with Fornell-Larcker’s criteria of 0.6 (Fornell and Larcker, 1981). All other indicators supported the reliability of the construct. Considering the results of the tests for reliability and validity, the constructs found to be used to investigate the conceptual model. After the validity and reliability test, the observed values of the items in each constructs were aggregated as an average to be used for regression analysis to test the hypotheses.

### Test of Hypotheses

The moderated mediation model was performed to test the postulated relationships among variables. The results are summarized in Table 2. Against our hypothesis that learning agility may be positively associated with innovative behavior, the direct path between learning agility and innovative behavior was found positive and significant (*B* = 0.56, *p* < 0.001, 95% CI [0.45, 0.68]). Thus, H1 was supported.

**TABLE 2:** Regression result for the moderated mediation model.

Predictor	Employee engagement				Innovative behavior			
	*B*	SE	*t*	95% CI	*B*	SE	*t*	95% CI
Consistent	–2.05	0.23	−9.13***	−2.50, −1.61	1.36	0.24	5.61***	0.88,1.83
Gender	–0.03	0.07	–0.45	−0.16, 0.10	–0.13	0.06	–2.05	−0.25, −0.01
Working years	0.02	0.04	0.45	−0.06, 0.09	0.03	0.04	0.73	−0.05, 0.10
Position	0.11	0.07	1.58	−0.03, 0.25	0.07	0.07	0.45	−0.06, 0.21
Organization size	0.02	0.04	0.42	−0.06, 0.09	–0.06	0.04	–1.65	−0.14, 0.01
LA	0.52	0.06	9.41***	0.41, 0.53	0.56	0.06	9.40***	0.45, 0.68
EE	−	–	−	–	0.34	0.06	5.58***	0.22, 0.46
POS	−	–	−	–	–0.07	0.06	–1.23	−0.18, 0.04
EE × POS	−	–	−	–	0.17	0.06	2.87**	0.06, 0.29
Mediation Effect*^a^*	–	–	−	−	0.18	0.04	−	0.10, 0.26
IMM*^b^*	–	–	−	–	0.09	0.03	−	0.04, 0.15
*F*	(5, 325) = 20.49***				(8, 322) = 31.46***			
*R* ^2^	0.24				0.44			

N = 331. Control variables include gender, working years, position, and organization size; 95% CI = Bootstrap confidence intervals with lower and upper limit; LA, learning agility; EE, employee engagement; POS, perceived organizational support; IB, innovative behavior; IMM, index of moderated mediation; ^a^Effect of learning agility on innovative behavior through employee engagement; ^b^Moderation effect of POS on mediation effect. **p < 0.01; ***p < 0.001.

**FIGURE 2:**
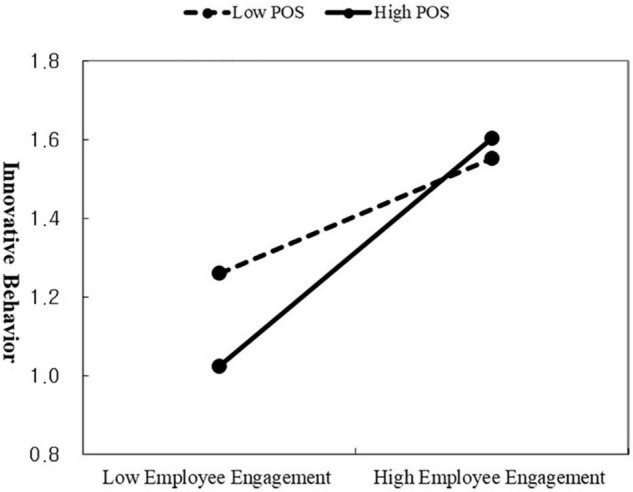
Moderating effect of POS on the relationship between employee engagement and innovative behavior.

Second, against our hypothesis that employee engagement would mediate the relationship between learning agility and innovative behavior, the indirect relationship between learning agility and innovative behavior, mediated through employee engagement, was found significant (*B* = 0.18, 95% *CI* [0.10, 0.26]). Hence, H2 was supported. Third, we had hypothesized that the relationship between employee engagement and innovative behavior would be moderated by POS. The interactions between employee engagement and POS were found to affect innovative behavior (*B* = 0.17, *p* < 0.01, 95% *CI* [0.06, 0.29]). We then applied the procedures of Aiken et al. (1991) to plot the pattern of significant moderation effect. High POS was set as one *SD* above the mean, while low POS was set as one *SD* below the mean. As depicted in Figure 2, the relationship between employee engagement and innovative behavior is positive for all two lines as indicated by their positive slope. Hence, higher levels of employee engagement accompany higher levels of innovative behavior. Due to the positive moderating effect, at high levels of moderator POS, the effect of employee engagement on innovative behavior is stronger, while at lower levels of moderator POS, that effect is weaker. Thus, the relationship between employee engagement and innovative behavior becomes stronger with high POS levels, which supports H3.

**FIGURE 3:**
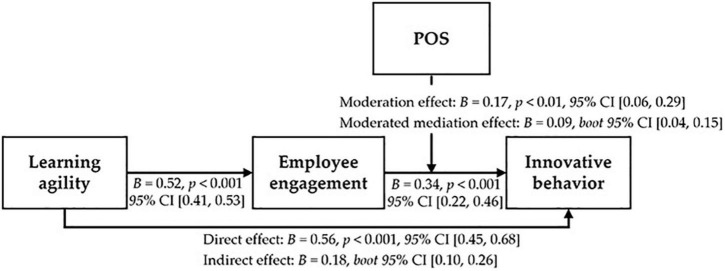
Results for the testing hypothesis.

Finally, we had hypothesized that the mediation effect, in which learning agility affects innovative behavior through employee engagement, would be moderated by POS. The result showed that the index of moderated mediation was significant (*B* = 0.09, 95% *CI* [0.04, 0.15]), verifying that the indirect effect of learning agility on innovative behavior through employee engagement is dependent on POS. Hence, H4 was supported (see Figure 3).

**FIGURE 4:**
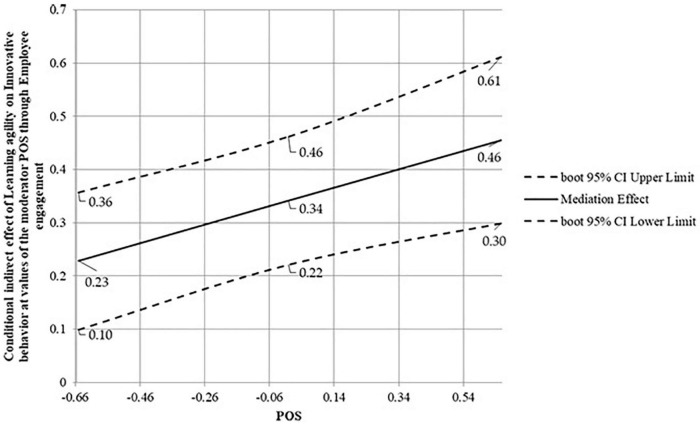
Conditional mediation effects of learning agility on innovative behavior at values of the POS.

The slope of the conditional indirect effect in Figure 4 is positive, meaning that the indirect effect of learning agility on innovative behavior through employee engagement is an increasing function of POS. Specifically, the conditional mediation effect of learning agility on innovative behavior through employee engagement was positive and significant at low (*B* = 0.23, *p* < 0.001, 95% *CI* [0.10 0.36]), moderate (*B* = 0.34, *p* < 0.001, 95% *CI* [0.22, 0.46]), and high (*B* = 0.46, *p* < 0.001, 95% *CI* [0.30, 0.61]) levels of POS, where low, moderate, and high were at the −1 SD (−0.66), Mean (0), and +1 SD (+0.66) of the POS mean-centered distribution, respectively.

Additionally, the models explained the considerable amounts of variance in innovative behavior (see Table 3). Control variables were able to explain only 5% of the variance in innovative behavior (*R*^2^ = 0.05). The addition of the learning agility and employee engagement variables led to a substantial increase in the amount of variance explained in innovative behavior (*R*^2^ = 0.42, Δ*R*^2^ = 0.37, *p* < 0.001), with both variables emerging as significant independent predictors. The variables in the regression equation in model 3 explained 44% of the variance in innovative behavior (*R*^2^ = 0.44, Δ*R*^2^ = 0.02, *p* < 0.01), with control variables, learning agility, employee engagement, POS, and interaction term. Thus, altogether, the variables under consideration were able to explain the largest variance in innovative behavior.

**TABLE 3:** Predicting innovative behavior.

Predictor	Model 1			Model 2			Model 3		
	*B*	SE	*t*	*B*	SE	*t*	*B*	SE	*t*
Consistent	3.59	0.08	44.70***	1.32	0.24	5.39***	1.36	0.24	5.61***
Gender	–0.22	0.08	−2.78**	–0.14	0.06	−2.17*	–0.13	0.06	–2.05
Working years	–0.12	0.05	–0.36	0.03	0.04	0.74	0.03	0.04	0.73
Position	0.21	0.09	2.36*	0.07	0.07	1.01	0.07	0.07	0.45
Organization size	0.01	0.05	0.11	–0.06	0.04	–1.47	–0.06	0.04	–1.65
LA	−	–	−	0.58	0.06	9.68***	0.56	0.06	9.40***
EE	−	–	−	0.27	0.05	5.04***	0.34	0.06	5.58***
POS	−	–	−	−	–	−	–0.07	0.06	–1.23
EE × POS	−	–	−	−	–	−	0.17	0.06	2.87**
*F*	(4, 326) = 4.21**			(6, 324) = 39.38***			(8, 322) = 31.46***		
*R* ^2^	0.05			0.42			0.44		
Δ *R*^2^	-			0.37***			0.02**		

N = 331. Control variables include gender, working years, position, and organization size; LA, learning agility; EE, employee engagement; POS, perceived organizational support. *p < 0.05; **p < 0.01; ***p < 0.001.

## Discussion

Employees’ innovative behavior (e.g., developing, adopting, and implementing new ideas for products and work methods) is an important asset that enables an organization to survive, sustain itself, and succeed in a dynamic environment (Kanter, 1983; Yuan and Woodman, 2010). This study aimed to evaluate how learning agility, employee engagement, and POS predict innovative behavior in the workplace. Based on the literature, we combined learning agility, employee engagement, and POS to construct the moderated mediation model describing the learning and motivational sources of employees’ innovative behavior. The results are as follows. First, we found that learning agility is positively related to innovative behavior. A learning-agile person is curious about the world and has a high tolerance for ambiguity, good skills, and vision (Gravett and Caldwell, 2016). According to the meta-analysis, these individual tendencies are related to innovation (Da Costa et al., 2015). Therefore, it is necessary to pay attention to learning agility, which acts as a prerequisite for developing innovative behaviors of organization members.

Second, employee engagement showed a significant mediating effect between learning agility and innovative behavior. These results are consistent with previous studies, suggesting that learning agility is related to employee engagement (Jeong and Sung, 2018; Saputra, 2018; Saputra et al., 2018; Kwon and Lee, 2020) and that employee engagement is motivated by innovative behavior (Agarwal, 2014; Gorgievski et al., 2014; Chen and Huang, 2016; Kim and Park, 2017). The componential theory on innovation describes how domain-relevant knowledge, a creativity-relevant process, and task motivation are simultaneously required to develop innovation for employees (Amabile, 1983). Learning allows us to acquire fundamental and important domain knowledge, information, and skills for innovation. Therefore, the employee’s learning agility plays an important role. In particular, high learning agility is associated with the tendency to find solutions to difficult problems, be comfortable with diversity and differences of opinion, and be very flexible and adaptable (De Meuse et al., 2010), which is considered a creativity-relevant process. Furthermore, employee engagement, a state of positive motivation for work, can be described as task motivation to develop employees’ innovative behavior.

Third, POS shows that learning agility has a moderated mediation effect on innovative behavior through employee engagement. Previous research that applied SET theory to innovative behavior found a moderating effect of POS on promoting employees’ innovative behavior (Zacher and Winter, 2011; Jain et al., 2013; Du et al., 2018). When employees receive particular resources from their organization, they feel obligated to respond with innovative behavior for the benefit of the organization (Ramamoorthy et al., 2005). POS also facilitates employee involvement through its supportive mechanism and strengthens the decision-making process related to innovative behavior (Cook and Wall, 1980; Choi et al., 2016). Furthermore, as uncertainty resulting from innovation is generally considered stressful, POS is obviously an important moderator (Vieitez et al., 2001). We confirmed the significant moderated mediation effect of POS on the psychological mechanisms that generate employees’ innovative behavior in this study. According to the JD-R, engagement affected by job and personal resources predicts extra-role performance as a positive result, and job demand controls the overall process (Schaufeli and Bakker, 2004). By applying this model, we have scientifically identified the complex and detailed process of generating innovative behavior by applying POS to the path to employees’ learning agility as a personal resource to achieve innovative behavior, a representative type of extra-role performance, through employee engagement.

### Theoretical and Practical Implications

We offer three theoretical implications of this research. First, we found significant positive associations between learning agility and innovative behavior, expanding the theoretical discussion of innovative behavior by empirically identifying its relationship with learning agility, as attempted by a few recent studies (Han, 2018; Kwon and Lee, 2020; Chu and Kim, 2021; Putri and Suharti, 2021). Second, although learning agility and employee engagement are considered important prerequisites for employees’ innovative behavior, there has been a lack of research on this topic. This study expands our understanding of the process in which innovative behavior is facilitated, and develops and identifies a new mechanism related to the relationship between learning agility and innovative behavior. Additionally, we empirically confirmed the importance of employee engagement, a key variable of physical and mental health, and learning agility for triggering innovative behavior, an important variable of an organization’s environmental innovation. Third, the study investigated the influence by comprehensively considering the factors suggested by the engagement model (JD-R model) and innovation theory (componential theory), unlike prior studies that have only partially studied the elements of innovative behavior.

Our findings also have managerial implications. First, members of an organization should develop learning agility to behave more innovatively, because learning agility not only builds emotional and cognitive engagement but also enhances effort and motivation for performance. Therefore, it is possible to promote the organization’s learning agility by identifying the specific characteristics of employees with high learning agility, establishing competency modeling, and developing and applying a competency-based curriculum. This would help develop an organizational culture that emphasizes the importance of learning agility by linking the company’s vision or mission with learning agility, and by configuring learning agility evaluation items in recruitment or promotion.

Second, the mediating effect of employee engagement on the relationship between learning agility and innovative behavior suggests that employee engagement and learning agility must be considered simultaneously to promote innovative behavior. Therefore, it is necessary to measure these characteristics regularly for systematic management and development of learning agility and employee engagement. Based on the measurement results, employees with relatively low levels of learning agility or employee engagement can be selected, and differentiated training/education programs or counseling can be provided. Managers need to help these employees set and perform goals for learning and work with initiative and responsibility by providing them with autonomy.

Third, we found that POS positively regulates the influence of learning agility on innovative behavior through employee engagement. Therefore, executives should understand that innovative activities need to be combined with organizational support. Executives may also set up tangible and intangible incentives to promote innovative behavior. Additionally, it is important to not only provide positive social recognition for innovative employees but also break the psychological comfort with the status quo and sensitize employees to opportunities for further improvement.

### Limitations and Recommendations for Future Research

Although the study serves as a useful baseline for further investigations, there are some limitations. First, it used a convenience sampling of employees in a few South Korean companies, relying on a self-reported survey, which might have led to sampling bias. Therefore, to increase generalizability, it would be better to consider a large sample with varied industrial characteristics and participant demographics in future studies. Furthermore, various measurement methods need to be applied to overcome the limitation of a self-reported survey. Second, we explored the relationship between learning agility and innovative behavior, and confirmed the mechanism to facilitate it. Future studies might explore further mechanisms by considering other related variables and include cross-level analyses at team, department, and organization levels. Third, our data were collected at a single time point. Longitudinal design can be used to provide insights into the changes in the variables over time and to effectively minimize common method bias.

## Conclusion

This study explored the mediation role of employee engagement and the moderation role of POS in the relationship between learning agility and innovative behavior. It explained when and how the learning characteristic relates to employees’ innovative behavior. These results concretize previous research by clarifying the mediation and moderation factors in the relationship between learning agility and innovative behavior. In this study, engagement was found to serve as a potential mediation mechanism between learning agility and innovative behavior in employees, and the mediation is moderated by POS. The relationship between learning agility and innovative behavior mediated by employee engagement appears to strengthen with higher levels of organizational support. Our findings demonstrate the importance of the moderated mediation model in understanding the mechanism linking learning agility and work-related innovative behavior in employees.

## Data Availability Statement

The raw data supporting the conclusions of this article will be made available by the authors, without undue reservation.

## Ethics Statement

Ethical review and approval was not required for the study on human participants in accordance with the local legislation and institutional requirements. The patients/participants provided their written informed consent to participate in this study.

## Author Contributions

YJ: methodology, validation, formal analysis, and writing—original draft preparation. AH: review, editing, supervision, project administration, and funding acquisition. Both authors conceptualization, read, and agreed to the published version of the manuscript.

## Conflict of Interest

The authors declare that the research was conducted in the absence of any commercial or financial relationships that could be construed as a potential conflict of interest.

## Publisher’s Note

All claims expressed in this article are solely those of the authors and do not necessarily represent those of their affiliated organizations, or those of the publisher, the editors and the reviewers. Any product that may be evaluated in this article, or claim that may be made by its manufacturer, is not guaranteed or endorsed by the publisher.
